# Research Progress of Molecular Simulation in Acrylamide Polymers with High Degree of Polymerization

**DOI:** 10.3390/molecules29112589

**Published:** 2024-05-31

**Authors:** Zhenye Liu, Qi Feng, Zhuangzhuang Xu, Shuangchun Yang

**Affiliations:** 1 College of Petroleum Engineering, Liaoning Petrochemical University, No. 1, West Section of Dandong Road, Wanghua District, Fushun 113001, China; wsymhgdyjs@163.com (Z.L.); 15668554951@163.com (Z.X.); yangchun_bj@126.com (S.Y.); 2 School of Civil Engineering, Liaoning Petrochemical University, No. 1, West Section of Dandong Road, Wanghua District, Fushun 113001, China

**Keywords:** molecular simulation, acrylamide polymer flooding, linear polymers, self-assembly, cross-linked polymers

## Abstract

Acrylamide polymers with a high degree of polymerization are widely used in petroleum production. It is of great significance to study the oil displacement mechanism of acrylamide polymers with a high degree of polymerization from the micro level. In recent years, the rapid development of computer molecular simulation technology has filed the gaps in macroscopic experiments and theories. This technology has been highly valued in the study of the molecular behaviour of polymer systems. In this paper, the research progress of molecular simulation applied to high-polymerization-degree acrylamide polymer is summarized. The application status of acrylamide polymer flooding, the analysis of polymer flooding mechanisms, and the research progress of molecular simulation in acrylamide linear and crosslinked polymers are expounded. Finally, the development prospect of acrylamide polymer research is given, and suggestions are put forward in terms of simulation direction and simulation tools.

## 1. Introduction

In recent years, the production of polyacrylamide (PAM) has reached millions of tonnes and has a wide range of applications in many fields. Especially in oil extraction [1], PAM has become a vital chemical additive because of its excellent performance. PAM is mainly used as a filter loss reducer, flocculant, diluent, plugging agent, etc., in oil extraction. It plays a vital role in dissection and water plugging, tertiary oil recovery technology [2,3], and drilling processes [4,5], which helps to improve the oil recovery rate. From 2018 to 2021, the market demand for polyacrylamide for oil and gas development in China grew from 499,000 tonnes to 526,000 tonnes. This growth trend reflects the steadily increasing demand for PAM in oil repellency as oil and gas extraction increases and recovery rates improve. Although much of the research on PAM is carried out in the laboratory, molecular modelling techniques can be a powerful tool. They can guide the selection of experimental parameters and help researchers design experiments more efficiently. In addition, molecular simulation can provide insights into the nature and behaviour of PAM, providing a theoretical basis and guidance for its application in emerging fields. Relevant market forecasts and investment strategy reports for 2023 may provide in-depth analyses of PAM market demand, industry growth, and investment opportunities. These reports are essential in order to understand the industry trends and formulate business strategies accordingly, and market demand is growing with the increase in oil and gas extraction. Molecular simulation is used as a research tool to help in the better understanding and application of PAM and to drive the development of its application in oil extraction as well as other emerging fields.

Molecular simulation is a cutting-edge computational technology that constructs diverse molecular models through computer simulation to study the structure and behaviour of molecules in depth. This technique is capable of revealing physical properties that are difficult to measure using experimental methods and has attracted great attention in recent years in the study of fluids, rock properties, and the analysis of related phenomena. It is not only an advanced research method, but also a powerful tool for in-depth analysis and deterministic decision-making in complex systems at the atomic and molecular levels. Rooted in the deep theoretical foundations of statistical mechanics and quantum mechanics, molecular simulation can accurately predict the equilibrium adsorption properties, kinetic behaviour, and thermodynamic properties of molecules by simulating and statistically analysing the trajectories of all the atoms in the system. This approach allows scientists to investigate the reaction mechanisms and physicochemical properties of a system through “computer experiments”, thus providing a valuable complement to and support for experimental and theoretical studies. With the development of molecular simulation, researchers now have more advanced tools to expand the boundaries of knowledge and advance science and industry.

Molecular modelling plays a crucial role in the study of reservoir fluid properties. Through this technique, researchers are able to deeply analyse the equilibrium and transport properties of reservoir fluids and simulate various fluid properties and their associated phenomena. It provides the observer with a unique view of the motion behaviour of molecules in the form of a 3D visualization, which greatly enhances the understanding of microscopic processes. Currently, numerous studies have begun to apply molecular simulation to consider higher levels of rock inhomogeneity, including pore scale, mineral composition, and more complex multi-component reservoir fluid systems. These advances have not only enriched the scope of molecular simulation applications but also hold great significance for the detailed characterization and in-depth analysis of hydrocarbon reservoirs. In particular, molecular simulation technology provides a unique solution when experimental conditions are limited or difficult to achieve. It can reveal the production mechanisms and flow-related phenomena of reservoir fluids, providing valuable insights for the oil and gas industry. These insights help optimize oil and gas recovery strategies, improve recovery rates, and provide a scientific basis for addressing the complex challenges in oil and gas reservoir development.

According to a market research report, the global polyacrylamide (PAM) market is estimated to reach a value of approximately USD 6 billion by 2022 [6,7]. As a water-soluble polymer, polyacrylamide plays a crucial role in the petrochemical industry, especially in the applications of water plugging adjusters, drilling fluid additives, tertiary oil recovery technology, and anti-scaling agents. In China, the demand for highly polymerized polyacrylamides as oil repellents is expected to continue growing as the difficulty of oilfield extraction continues to rise and environmental regulations become increasingly stringent. In order to seize this market opportunity, domestic enterprises are increasing their R&D efforts and are committed to improving the quality and performance of their products in an attempt to gradually replace imported products. In addition, high-polymerization polyacrylamide exhibits excellent salt and temperature resistance properties, enabling it to operate stably in high mineralization and high-temperature environments. This provides great potential for its application in the development of high mineralization and high-temperature oilfields. However, despite the promising prospects for the application of polyacrylamide in the field of oil repulsion, there is still a gap to be bridged between domestic producers in the field of medium- and high-end products and international giants in the chemical industry such as Aisen (SNF) and BASF. In order to improve the economic efficiency of the domestic polyacrylamide industry and enhance its competitiveness in the global market, local enterprises urgently need to promote the transformation of products from low-end to high-end markets.

In addition to its use in oil extraction, polyacrylamide has important applications in a number of other fields. As a flocculant, polyacrylamide is widely used in municipal wastewater treatment, industrial wastewater treatment, etc. It can effectively separate solids and liquids and promote sludge dewatering. Polyacrylamide also has applications in the biomedical field, for example, as a slow-release material for certain drugs or in bioassay technology. In the metallurgy and mineral processing industry, polyacrylamide is used as a flocculant and dispersant to improve the beneficiation efficiency of ores. In agriculture, the demand for highly absorbent resins is growing year by year, and polyacrylamide can be used in forestry and agriculture to retain water and improve the water retention capacity of the soil.

To sum up, the prospect of polyacrylamide application in the field of oil displacement is considered positive and optimistic, but it also faces a series of challenges. These challenges include improving product quality, enhancing performance, and realizing high-end products. Domestic enterprises need to respond to these challenges and seize the opportunities of industry development through technological innovation and market strategy adjustment. This paper gives an overview of the progress of computer molecular simulation technology applied to the study of high polymerisation acrylamide, gives an account of the current status of the application of acrylamide polymers for oil repulsion, an analysis of the polymer repulsion mechanism, a study of molecular simulation in acrylamide linear and cross-linked polymers, and then finally gives an outlook of acrylamide polymers for oil repulsion.

## 2. Current Status of Application of Acrylamide Polymers for Oil Repulsion

Polymer oil drive technology is a method to enhance the viscosity of the replacement fluid by adding polyacrylamide (PAM) to the injection water. This viscosity enhancement can effectively reduce the formation water content to 50% and enhance the drag coefficient by 5–15%, optimising the oil-to-water flow ratio in the reservoir, thus significantly improving the macro-replacement efficiency and crude oil recovery [8,9,10,11]. The technology originated in the United States in the mid-20th century, and the first field test was successfully conducted in 1964. In China, the exploration of polymer drive technology was synchronised with the discovery of the Daqing oilfield and started in the 1970s. As a large onshore sedimentary sandstone oilfield, the Daqing oilfield has an oil layer of approximately 1000 m depth and a reservoir temperature of approximately 45 °C. The mineralisation of the original formation water is approximately 7000 mg/L, whereas that of the injected water ranges between 400 mg/L and 1000 mg/L [12]. Since the 1970s, Daqing Oilfield has carried out a number of tests, including polymer injection tests during the exceptionally high water content period in the SII7+8 layer of the Xiaoyingdiannan well group, as well as single and double layer polymer oil repulsion tests in the thick layer test area, the western part of the Central Zone, and the industrial polymer tests in the North I section, all of which have achieved remarkable results in reducing water and increasing oil. Especially between 2002 and 2006, Daqing Oilfield achieved a stable annual oil production of more than 1000 × 10^4^ tonnes for five consecutive years with the help of polymer FD technology [11,12]. The oil drive technology adopted in Daqing Oilfield is “alkali/surfactant/polymer” ternary complex drive technology, with alkylbenzene sulfonate as the main agent. Meanwhile, Shengli Oilfield applied the binary composite drive technology of “surfactant/polymer” [11]. With the continuous maturity and improvement of the technology, Dagang Oilfield, Henan Shuanghe Oilfield, and Liaohe Oilfield have also adopted the polymer flooding technology and have achieved good effects in water reduction and oil increase [12].

Polyacrylamide (PAM) shows excellent performance with its high degree of polymerisation and has higher molecular weight and molecular mass, which makes its adsorption capacity on the surface of rocks and oil droplets significantly enhanced, and thus effectively improves the recovery of crude oil. In view of this, the application of high-polymerization PAM in oil displacement technology has become a valuable and far-reaching research field. The in-depth study of the conditions and key factors influencing polymerization reactions is expected to open up new paths and strategies for the enhancement of crude oil recovery. In addition, this research cannot only promote the efficiency of oil extraction but also contribute to environmental protection and sustainable development. Therefore, it is of great significance to use molecular simulation techniques to explore the high polymerisation acrylamide polymers in depth [9]. Through molecular simulation techniques, it is possible to predict and optimise the physicochemical properties of highly polymerized materials, such as the mechanical properties, thermal stability, and solubility. This helps to predict properties at the material design stage, reducing the number and cost of experiments. Moreover, molecular simulation techniques allow the study of compatibility between different polymers and the interaction of polymers with other molecules, which is important for the development of new composite materials and the improvement of material properties. Theoretical simulation studies on the multilevel aggregation state structure of polymers are of great significance in understanding the relationship between the macroscopic properties and microstructure of materials, and they also help in the development of new polymer materials. At present, research on the oil-repelling effect of high-polymerization PAM is mostly focused on the laboratory environment. Molecular simulation technology can assist in the precise selection of experimental parameters, greatly improving the efficiency of experimental design and the accuracy of experimental execution. At the same time, molecular simulation provides researchers with an in-depth analysis of the properties and behaviours of PAM, which can help reveal its potential applications in a number of emerging fields. Guided by molecular simulation, researchers can more precisely understand the mechanism of action of high-polymerization PAM in oil–water systems and how its performance can be enhanced through the optimization of molecular structure. This will not only facilitate the transition from laboratory research to industrial application of high-polymerization PAM oil-repellent technology but will also bring revolutionary advances in the field of oil extraction.

## 3. Application of Molecular Modelling Techniques to Different Acrylamide Polymers

Molecular simulation, a cutting-edge tool in the field of computational chemistry, is dedicated to the in-depth investigation of physicochemical phenomena at the molecular level. It provides deep insights into understanding and predicting molecular behaviour by accurately simulating intermolecular interactions. Molecular simulation plays an irreplaceable role in the study of acrylamide polymers across a wide range of applications. This technology provides researchers with valuable insights by revealing the molecular mechanisms of acrylamide polymers in different application scenarios. It not only assists scientists in understanding the performance characteristics of polymers but also provides strategic guidance in the development of new materials, performance optimization, and process improvement. The application of molecular simulation technology has greatly advanced the research of acrylamide polymers in a variety of fields, such as water treatment, oil extraction, the paper industry, and biomedicine. Through simulation, researchers are able to predict the reactions and effects of polymers in specific environments without the need for actual experiments, thus effectively shortening the R&D cycle, reducing R&D costs, and accelerating the translation of innovations. With the continuous improvement of computing power and optimization of simulation algorithms, the application of molecular simulation technology in the research of acrylamide polymers will become more extensive, and its potential will be further realized, bringing more far-reaching impacts on scientific research and industrial applications.

Molecular dynamics (MD) simulation is a computational chemistry method for studying the dynamic behaviour of molecular systems. It predicts the trajectory of an atom or molecule at a given time by solving the Newtonian equations of motion. MD simulations have a wide range of applications in several fields, including, but not limited to, biology, chemistry, materials science, and physics. It also has a range of advantages and limitations. MD simulations provide detailed information at the atomic level, allowing intermolecular interactions and motions to be observed. At the same time, it can cover time scales from picoseconds to milliseconds, allowing the study of fast or slow dynamic processes that can predict the structural, energetic, kinetic, and thermodynamic properties of molecular systems. Although MD simulations can cover a wide range of time scales, current computational capabilities are still limited for certain very slow processes (e.g., protein folding), while MD simulations typically require significant computational resources, including high-performance computing clusters and large amounts of storage space. This article lists some of the methods of simulation, and their advantages and disadvantages can be seen in Appendix A (Table A1).

In MD simulations, periodic boundary conditions are often used, so the system size should be chosen to ensure that there are no unrealistic interactions within the simulation box. In MD simulation, the main focus is to analyse the trajectory of atoms or residues to understand the process of protein folding, ligand binding, etc. The root mean square deviation (RMSD) between the reference structure and the simulated structure is calculated to assess structural changes. The simulation results are eventually combined with experimental data to validate and interpret the simulation findings.

### 3.1. Analysis of Polymer Driving Mechanisms

Polymer oil driving technology is a technology that uses water-soluble polymers to increase the viscosity of water and use it as an injector for oilfield development, which can improve oil recovery efficiency. Through oil displacement, the oil–water flow ratio can be improved, which can then slow down the rise of water content in the extracted mixture. This makes the oil displacement efficiency closer to the limit of oil displacement efficiency, which is a key technology for improving the efficiency of oil displacement work.

The flow of non-Newtonian fluids in porous media exhibits high complexity, which covers several key factors such as nanoscale confinement effects, interfacial interactions, and fluid viscoelasticity [13,14,15]. In oilfield development, atomic-level interfacial interactions between hydrocarbons and repellents (e.g., ultra-high molecular weight polyacrylamides, polyvinyl alcohols, etc.) and the elasticity and viscosity of the repellents are critical for enhancing the recovery of residual oil. An effective oil drive strategy requires that the repellents have sufficient “power” to overcome the interaction forces between the oil and the pore wall in the pore space of the chalk sand or under special conditions. Wang et al. [16] from the Daqing Oilfield Company found that when elastic fluid passes through the “dead zone” of the reservoir, in addition to generating shear stress it also creates a positive stress between the oil and the flowing fluid, and this traction effect can help to displace the residual oil in the dead zone. In addition, for the polymer solution at high speed through the porous medium, due to the elastic properties of the polymer chain, shear thickening phenomenon will occur [17]. Clarke et al. [18] observed a significant shear thickening effect by measuring the rheological properties of polymer solutions in a microfluidic network, a phenomenon attributed to the formation of elastic turbulence. Clarke et al. also used nuclear magnetic resonance diffusion measurements to investigate the flow behaviour of the residual oil in a complex three-dimensional structure during polymer oil removal and found that the generation of elastic turbulence within the flowing polymer solution may contribute to the upgrading of the polymer oil. Elastic turbulence may play a positive role in enhancing oil recovery [19]. Clarke et al. carried out an in-depth study on the oil film adsorbed on the rock surface, and the results showed that the velocity gradient of the elastic fluid near the wall was much larger than that of the Newtonian fluid, which resulted in a stronger force for oil film detachment [20,21].

Recent research advances have significantly helped to deepen our understanding of polymer oil drive mechanisms. Although most of these studies have focused on oil recovery processes at the micron level, they have laid the foundation for subsequent studies at the molecular level. Some scholars have begun to shift their research focus to the molecular simulation of polymer-assisted oil displacement for a deeper understanding. By studying the phenomenon of local aggregation of NPAM molecular chains in the polymer solution system, Xu [22] revealed the interaction process of multiphase molecules during polymer flooding, quantified the diffusion law of three kinds of molecules (polymer, oil, and water), and enriched the theoretical system of polymer flooding flow.

**Figure 1:**
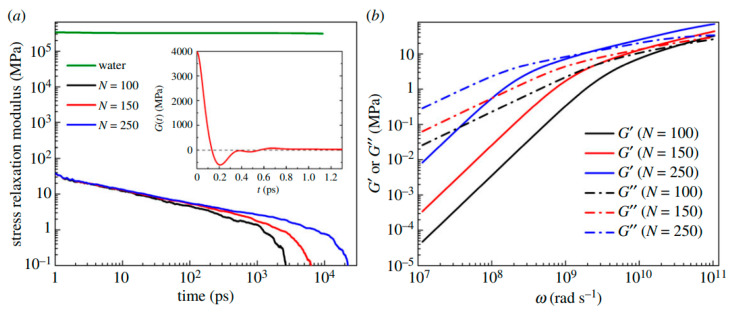
(**a**) Stress relaxation modulus G(t) for water and polymers with chain length N = 100, 150, and 250. The inset illustrates the same data for N = 100 short time-scale fluctuations at an early time arising from bond interactions. (**b**) Storage modulus G(ω) and loss modulus G(ω) for different chain length polymers calculated by Maxwell modes fit to the data of G(t). Solid and dash–dot lines denote the storage modulus and the loss modulus, respectively [23].

Fan et al. [23] from the University of Science and Technology of China (USTC) thoroughly investigated the dynamic process of the dead-end displacement of trapped oil droplets in nanopores through MD simulations. Their study highlighted that the key to the excellent oil recovery achieved by polymer oil repulsion lies in the unique elastic properties possessed by the polymer molecules. In addition, they carefully analysed the viscoelasticity of polymers and found that the length of the polymer chain is directly proportional to its storage modulus, and the longer the chain length, the longer the relaxation time of the polymer, which makes the elasticity effect of polymers even more significant. Figure 1a illustrates the G(t) curves for water and polymers with varying chain lengths. Water’s G(t) curve is flat, signifying that it does not exhibit stress relaxation characteristics. This suggests that water is not classified as a viscoelastic fluid. In contrast, polymers display evident non-Newtonian stress relaxation behaviour, implying that, at a constant stress level, the stress gradually diminishes across multiple magnitudes. The outcome of this transformation is depicted in Figure 1b. The storage modulus signifies the energy retained due to elastic deformation, indicative of the polymer’s elasticity. Conversely, the loss modulus denotes the energy that is converted to heat during deformation, indicative of the polymer’s viscosity [23]. These findings will further deepen our understanding of the mechanism of polymer oil repulsion and provide a solid theoretical basis for the efficient production of oilfields.

Zhang [24] proposed a novel strategy for creating superwetting polymer filtration membranes. This approach leverages the synergistic effect of additive-induced micro/nano hierarchical structures and in-situ hydrolysis during the phase inversion process. Consequently, a superhydrophilic and underwater superoleophobic polyacrylonitrile (PAN) membrane was successfully fabricated. This PAN membrane demonstrated ultralow oil adhesion, which endows it with exceptional oil/water separation capabilities. It can effectively separate oil-in-water emulsions with a high permeation flux of up to 2270 L/m^2^·h·bar and an extremely high separation efficiency (oil residuals in the filtrate after one-time separation are lower than 10 ppm). Additionally, the membrane showed excellent recyclability during the oil-in-water separation process. The superior oil/water separation performance of the superwetting PAN membrane indicates its great potential for treating oily wastewater.

In summary, experimental research can analyze the oil repulsion mechanism of polymers, and molecular simulation can also provide effective assistance to the study of the polymer oil repulsion mechanism.

### 3.2. Molecular Simulation Techniques Applied to Linear Polymers of Acrylamide

Molecular simulation plays a crucial role in the field of acrylamide linear polymer research. As a state-of-the-art computational method, molecular simulation greatly advances the in-depth understanding of polymer structure and properties through the detailed computer simulation and prediction of molecules and their interactions. The technique is capable of monitoring and analysing conformational changes in polymer chains in real time, thereby revealing key physical properties such as polymer flexibility, rigidity, and chain conformation.

Through molecular simulation, researchers are able to observe the behaviour of polymers at the atomic level, thereby predicting material properties at the design stage and optimizing polymer synthesis processes. This computationally driven research approach not only improves research efficiency but also reduces experimental costs and accelerates the transition of new materials from the laboratory to industrial applications.

#### 3.2.1. Acrylamide Homopolymer of Molecular Structure, Rheological Properties, and Application to Oil Drive Efficiency Studies

**Figure 2:**
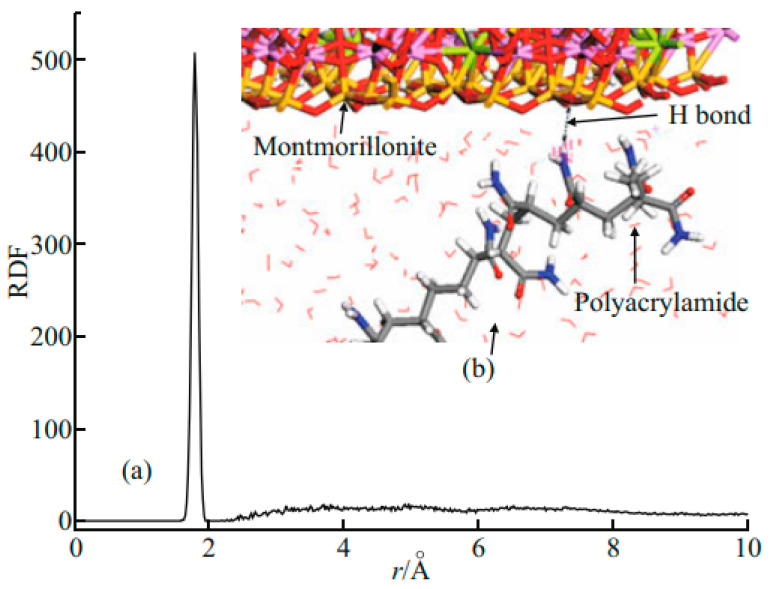
(**a**) Radial distribution function of the distance between hydrogen atoms of amide groups in polyacrylamide and oxygen atoms on the surface of montmorillonite clay layers. (**b**) The scheme of the H-bond interaction between the polyacrylamide and montmorillonite clay layer [27].

Acrylamide homopolymers, such as polyacrylamide (PAM), are polymeric materials made from the polymerisation of a single acrylamide monomer, possessing a unique chain structure and properties such as viscoelasticity and the ability to form latex films. Moud [25] investigated the effect of polymeric flocculants (especially nonionic polyacrylamide, PAM) on floc formation and settling behaviour. In the absence of added PAM, the interactions between the flocs were weak, resulting in their faster settling at the bottom of the vessel. On the contrary, when PAM was added, the PAM molecules formed bridges between the flocs, which enhanced the interconnections between the flocs and thus slowed down their settling rate. In order to assess the size and morphology of the flocs, several microscopic techniques were used in the study, including scanning electron microscopy (SEM) and transmission electron microscopy (TEM). These techniques were able to provide detailed images of the microstructure and morphological distribution of the floc surface. The results of SEM and TEM analyses showed that when PAM was not used the floc structure was looser and the sediment distribution was not uniform. This may be due to the lack of a sufficient bridging effect, resulting in poor binding between the flocs. However, when PAM was added to the flocs, SEM and TEM images showed that the floc structure became denser and more uniform. This suggests that the addition of PAM enhances the stability and structural tightness of the flocs, which in turn affects their settling characteristics. In conclusion, Moud’s study indicates that nonionic polyacrylamide (PAM), as a polymeric flocculant, is able to significantly alter the settling behaviour and structural properties of flocs by forming bridges between the flocs. The changes in the morphology of the flocs can be visualized through the application of microscopic techniques such as SEM and TEM to assess the effect of PAM on the flocculation process. Chen et al. [26] from Sichuan University investigated the structural properties and viscosity behaviour of PAM solutions at different NaCl concentrations by molecular dynamics simulations. The simulation results showed that the characteristic viscosity, radius of gyration, and hydrodynamic radius of the PAM solution did not change significantly under varying NaCl concentration conditions. Subsequently, Chen et al. [26] further explored the structure and characteristic viscosity of HPAM solutions at different NaCl mass fractions by computer molecular simulation in 2012, and they found that the characteristic viscosity of HPAM solutions, as well as its radius of gyration and hydrodynamic radius, decreased with the increase in salt mass fraction. In fluid mechanics, the hydrodynamic radius (also known as the Stokes radius or hydrodynamic radius) is a parameter that characterizes the resistance that molecules experience as they move through a fluid. A molecular model needs to be constructed that places the molecules in a simulated solvent environment, which can either be explicit, i.e., contain all solvent molecules, or implicit, where the effect of the solvent is approximated by a continuous medium model. The molecules are allowed to move freely at a given temperature and pressure through molecular dynamics (MD) simulations. During the simulation, the trajectories of the molecules are recorded. According to Stokes’ law, the resistance to the motion of a spherical particle in a viscous medium is proportional to its radius. In the simulation, the hydrodynamic radius of a molecule can be estimated by calculating its resistance. Li et al. [27] from Wuhan University of Technology investigated the effect of hydrated montmorillonite on the variation of PAM viscosity with temperature and shear rate under restricted shear conditions using regular systematic molecular simulation. The simulation results show that the radius of gyration of PAM decreases with increasing temperature or shear rate at higher shear rates. This phenomenon is attributed to the hydrogen bonding between PAM and the clay layer, and these hydrogen bonds have a significant effect on the stretching or bending behaviour of the PAM chains. The weaker hydrogen bonding interactions between the clay layer and PAM contribute to a more curled state of the PAM polymer chains, which leads to a decrease in their radius of gyration. Figure 2 shows that one sharp peak with the stronger intensity appears at approximately 1.9 Å. The sharp peak corresponds to the distance between H atoms of amide groups of PAM and oxygen atoms on the surface of clay, which is just within the range of H-bond length. These findings provide researchers with a deep understanding of the changes in the characteristic viscosity of PAM at the molecular level and open up new avenues for research related to the improvement of oil drive efficiency.

#### 3.2.2. Acrylamide Copolymer

Acrylamide copolymers are a class of polymers obtained by copolymerizing acrylamide with other monomers in a way that allows the properties of the polymer to be tailored to the specific needs of the application. A common acrylamide copolymer is Polyacrylamide (PAM). Polyacrylamide is a linear polymer prepared by the polymerization of acrylamide monomers. Polyacrylamide is widely used as a flocculant in the field of water treatment. In wastewater treatment and water purification, PAM improves water clarity and helps remove suspended solids and organic matter. In addition, the polyacrylamide can be used in specific water treatment applications by crosslinking to form a gel, such as for preventing water infiltration in oil field water treatment. In particular, copolymers of polyacrylamide with sodium polyacrylate and copolymers of acrylamide with hydrophobic monomers have garnered increased attention in molecular simulation studies. These studies have simulated the behaviour of copolymer molecules under different environmental conditions through computer simulation techniques, revealing the relationship between their microstructures and macroscopic properties. In recent years, with the enhancement of computational power and the continuous progress of simulation technology, the application of computer molecular simulation in the study of acrylamide polymers has become more and more widespread, which has aroused great interest among researchers. Through molecular simulation, researchers are able to observe and analyse the mechanism of action of acrylamide copolymers at the atomic level in industrial processes such as oil extraction. This not only deepens the understanding of the factors influencing the properties of copolymers but also provides a solid scientific basis for optimizing their properties through molecular design. The results of these molecular simulations are of great practical significance in guiding the synthesis of more targeted acrylamide copolymers and improving their efficiency in applications such as oil extraction. Through simulation, researchers can predict the performance of copolymers in practical applications and optimize their chemical structures to enhance their performance in specific environments, such as improving recovery, reducing costs, and minimizing environmental impact. With the continuous development of molecular simulation technology, it is expected that it will play an even more important role in the design and application of acrylamide copolymers.

##### Molecular Simulation Study of Copolymerisation of Polyacrylamide and Sodium Polyacrylate

**Figure 3:**
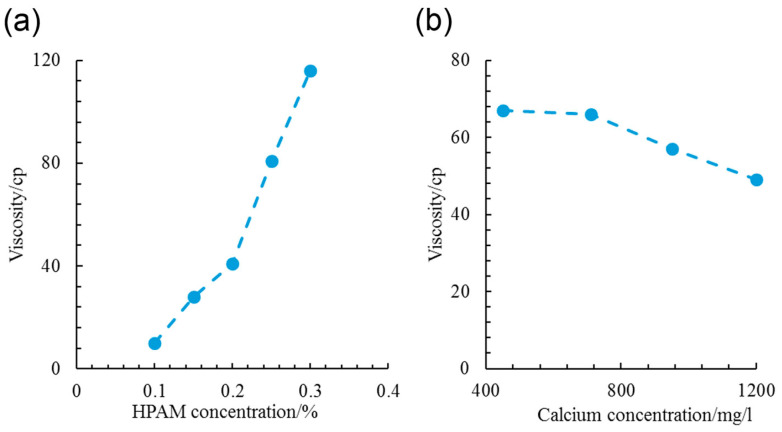
(**a**) HPAM concentration and (**b**) calcium concentration vs. measured apparent viscosity (all experiments in (**a**,**b**) were carried out under 333 K and 0.1 Mpa; the formation-water used in (**a**) was composed of 1.5 g/L sodium and 1.2 g/L calcium ions; HPAM and sodium concentrations of (**b**) are 0.2% and 1.5 g/L, respectively) [29].

The copolymer of sodium polyacrylate and polyacrylamide, known as hydrolysate of polyacrylamide (HPAM), has received much attention in the research field of molecular simulation techniques. Partially hydrolysed polyacrylamide (HPAM) solution is a commonly used method to improve oil recovery efficiency during tertiary oil recovery in oil fields. However, due to some limitations of indoor water flooding experiments, such as the limitation of application scope and too many assumed conditions, it is difficult for these experiments to deeply reveal the deep mechanism of polymer water flooding. To overcome these limitations, Xu [28] developed an interaction model and investigated polymer water flooding from a microscopic perspective. It was shown that the fluidity of HPAM solutions decreases with increasing HPAM concentration and molecular chain length, as well as the appropriate degree of hydrolysis. HPAM molecular chains can change from an elastically deformed state to a stress-hardened state when subjected to stress, which further affects the rheological properties of the solution. In addition, HPAM molecules can be adsorbed on the surface of minerals such as kaolinite, and this adsorption can increase the viscosity and decrease the permeability of water, thus playing an important role in tertiary oil recovery in oil fields. Through Xu’s [28] research, we are able to have a deeper understanding of the mechanism of HPAM’s role in tertiary oil recovery, which provides an important theoretical basis for optimizing polymer oil drive technology. Although the molecular simulation study of polyacrylamide and sodium polyacrylate copolymer once encountered a bottleneck, in 2021 Yang et al. [29], from the Sinopec Research Institute of Exploration and Development, made new progress through molecular dynamics simulation methods. They investigated the conformation and network structure of HPAM molecules in highly mineralized formation water and the viscosity changes from the microscopic dense phase to the dilute phase, and they verified these findings with experimental results. It was found that the molecular network structure in the microscopic dense phase plays a decisive role in the apparent viscosity of HPAM solutions, and the intervention of calcium ions disrupts this network, leading to a significant decrease in apparent viscosity. As shown in Figure 3, it can be seen that the measured apparent viscosity is contingent upon the gradients of HPAM and calcium concentrations. As the HPAM concentration escalates by a factor of 2–3, the measured apparent viscosities experience a substantial increase, ranging from 4 to 10 times. This observed change coincides with the simulation results of Yang’s team [29]. Upon delving into the simulation analysis, it becomes evident that the escalating apparent viscosity is a direct consequence of an augmented number of molecular network interactions within the dense phase. In addition, Ahsani et al. [30] from Semnan University, Iran, investigated the adsorption process of HPAM on rock surfaces using molecular dynamics (MD) simulations. They analysed the effect of HPAM on the wettability of carbonate reservoirs from a microscopic point of view and calculated the relevant energies and surface tensions of the three-phase system consisting of polymers, water molecules, and rocks. The simulation results show that the adsorption contact angle of HPAM decreases significantly at ambient and reservoir temperatures, indicating a shift from an oleophilic surface to a water wettability. The effect of temperature on the contact angle of HPAM adsorbed on the rock surface was also examined, and the results showed that the polymer adsorption on the rock surface increased with increasing temperature, and the surface wettability tended to be towards water.

##### Molecular Simulation of Copolymerisation of Acrylamide with Hydrophobically Modified Monomers

Molecular simulation plays an important role as a key research tool within the field of chemistry and physics. Through computer simulation, we can investigate the behaviour of the copolymerization of acrylamide with hydrophobically modified monomers in depth at the atomic level, simulate the structure and properties of the system, and study the microstructure, stability, and kinetic properties of the copolymer in detail by adjusting the simulation parameters. Ni et al. [31] from Sichuan University conducted a study using dissipative particle dynamics (DPD) simulations to investigate the hydrophobic association of three fluorocarbon-modified polyacrylamide copolymers: P(AM-AANa-3F), which incorporates trifluoroethyl methacrylate; P(AM-AANa-6F), which includes hexafluorobutyl methacrylate; and P(AM-AANa-12F), which embeds dodecafluoroheptyl ester. These copolymers were analysed at a mesoscopic level, and it was determined that P(AM-AANa-12F) demonstrated superior heat, shear, and salt resistance compared to the other copolymers. Sun [32] conducted a study to investigate the effect of different surfactants on the viscosity of two polymers of similar molecular weight: partially hydrolysed polyacrylamide (HPAM) and hydrophobically modified polyacrylamide (HMPAM). The study was conducted under conditions that simulate reservoir environments to assess how surfactants affect the rheological properties of these polymers. In addition, the study examined the potential impact of synergistic effects between surfactants and polymers on improving oil displacement efficiency. The experimental results showed that, for polymers such as HPAM, the surfactant reduces the viscosity of its solution mainly through an electrolyte-like effect. This viscosity-reducing effect contributes to the oil displacement efficiency of the polymers in the reservoir. As for HMPAM, although it is not explicitly mentioned in the paper, it can be hypothesized that the surfactant may affect its performance through a different mechanism, which may involve altering the interaction between the polymer and the reservoir rock. Through this study, Sun revealed the important role of surfactants in improving the oil repelling efficiency of polymers, especially in reducing the viscosity of HPAM, which provides valuable insights for optimizing chemical repelling technologies in oilfields. In the same year, Yuan et al. [33] from Shandong University investigated the Coulombic and coordination interactions between HM-HPAM and multivalent cations through molecular dynamics simulations, which significantly affected the polymer solution properties. Deng et al. [34], on the other hand, investigated the rheological properties of hydrophobically modified acrylamide copolymers (HA-PAMs) in saline solution, systematically examining the effects of polymer concentration, salt solution concentration, temperature, and shear rate on the rheological properties of the solution. The molecular behaviour of the polymer was described in detail using dissipative particle dynamics (DPD) mesoscopic simulation. It was found that the aggregation of hydrophobic groups formed hydrophobic domains, and the hydrophobic interaction between the side chains increased with the increase in shear rate, which promoted the aggregation of the polymers and thus enhanced the aggregation of hydrophobic side chains. In addition, the study explored the effect of salt on the molecular behaviour of HA-PAM from a microscopic point of view. On the one hand, the shielding effect of the electrolyte led to the curling of the macromolecules, which reduced the intermolecular entanglement and led to a decrease in the solution viscosity; on the other hand, the intermolecular hydrophobicity of the intermolecular aggregation was strengthened with the increase in salt concentration, which further led to the decrease in the solution viscosity, which was attributed to the shielding effect of the inorganic salt.

These findings not only improve our understanding of the behaviour of modified polyacrylamide copolymers under different conditions but also provide an important scientific basis for optimizing the properties of the polymers. This can help in developing more efficient and stable polymeric materials to meet the stringent requirements in industrial applications.

##### Molecular Simulation Study of Copolymerisation of Acrylamide with Other Monomers

**Figure 4:**
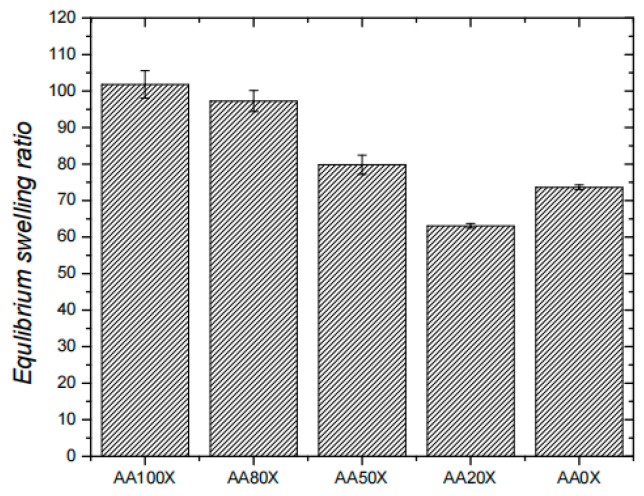
Equilibrium swelling ratio of pIPNs/magnetite composites [36].

Molecular simulation studies play a key role in the field of the copolymerization of acrylamide with other monomers, mainly focusing on exploring the interactions between monomers, the mechanism of copolymerization reactions, and the prediction of copolymer properties. Using molecular simulation techniques, researchers can gain insights into the chemical reaction processes between acrylamide and other monomers at the atomic level, optimize copolymerization conditions, and accurately predict the microstructure and macroscopic properties of copolymers. Seung et al. [35] from Pusan University in South Korea used molecular simulation methods to study in depth the swelling behaviour of the surface-grafted thermosensitive material poly(N-isopropylacrylamide). It was found that the material exhibited significant swelling characteristics below the critical solubility temperature, while the phenomenon of dissipative swelling occurred above the critical solubility temperature. Novel composite hydrogels based on poly(acrylic acid-co-acrylamide)/polyacrylamide pseudo-interpenetrating polymer networks (pIPNs) and magnetite were prepared via in situ precipitation of Fe^3+^/Fe^2+^ ions within the hydrogel structure. The aim of the study was to evaluate the role of P(AA-co-AAM)/PAAM pIPNs in the in situ formation of magnetite particles and therefore obtain novel P(AA-co-AAM)/PAAM pIPNs/magnetite composite hydrogels. Simeonov [36] assumed that the pIPNs’ composition and structure allow the control of the overall polymer network density and functionality, and hence they are expected to influence the size and size distribution of the magnetite particles deposited in situ when compared to the single PAA and PAAM networks. By comparing with traditional binary compound flooding, the binary system with nanoparticles has higher application value in enhanced oil recovery (EOR). The in situ formation of magnetite particles in the IPNs results in a significant increase in the ESR, and the obtained pIPNs/magnetite composites swell much more than the neat IPNs (Figure 4). In this work, Liu [37] prepared a flexible polymeric brushes hybrid nano-silica star-like hydrophobically associative polyacrylamide (SHPAM). Subsequently, a surfactant named alkyl alcohol polyoxyethylene ether sulfonate (CEOS) was selected to study the physicochemical properties of nanofluids through a combination of experiments and simulations. The results indicated that the prepared nanofluids had good dispersion stability in strong brine.

### 3.3. Studies in Polymer/Surfactant Self-Assembly

**Figure 5:**
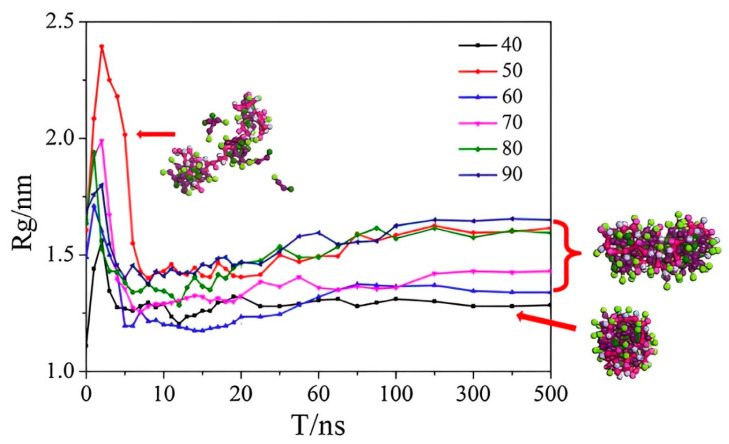
Radius of gyration (nm) of the polymer chain as a function of simulation time at different concentrations of DTAB (N_DTAB_ = 40, 50, 60, 70, 80, and 90) and the corresponding morphologies of aggregates [43]. (N_DTAB_ indicates the number of the DTAB).

The compounding technology of surfactants and polymers can trigger synergistic effects, which can significantly enhance the comprehensive performance of the composite system. With the introduction of molecular simulation techniques, the study of the self-assembly behaviour of polymers/surfactants has penetrated to the microscopic level, providing new perspectives for understanding their complex interactions and self-assembly mechanisms [38,39,40,41]. In such hybrid systems, a variety of ordered structures including spheres, rods, and vesicles can be formed. Wang et al. [42] from Shandong University explored the self-assembly process of polyacrylamide (PAM) and sodium dodecyl sulphate (SDS) in aqueous solution using coarse-grained molecular dynamics simulations. The simulation results showed that the PAM molecular chains tended to adsorb at the junction of the hydrophobic and hydrophilic regions of the SDS micelles, forming a unique “necklace” structure, which was attributed to the hydrophobic interactions between the polymer backbone and the hydrophobic tails of the surfactant. Hu et al. [43] from the China University of Petroleum (East China) investigated the self-assembly behaviour of a mixture of dodecyltrimethylammonium bromide (DTAB) and hydrolysed polyacrylamide (HPAM) using coarse-grained molecular dynamics (CGMD) simulations in conjunction with the Martini force field. The study systematically investigated the effects of DTAB concentration and the degree of hydrolysis of HPAM on the self-assembly structure and formation process. It was found that, under different hydrolysis degrees, the agglomerates gradually transformed from spherical to rod-shaped with the increase in DTAB concentration, and the rod-shaped structure tended to bend at high hydrolysis degrees. By calculating the relative shape anisotropy and radius of gyration (Rg), the authors quantitatively analysed the effect of DTAB concentration on the transition from spherical to rod-like morphology. The simulation results revealed that the steric hindrance effect due to the increase in DTAB concentration has a significant impact on the self-assembled morphology. To gain insights into the aggregation process, Hu’s team calculated the radius of gyration (Rg) of the HPAM chain as a function of simulation time. The results are presented in Figure 5, which provides a visual representation of the temporal evolution of the Rg values. This analysis is crucial for comprehending the structural dynamics and the formation mechanism of the aggregates. At lower concentrations of DTAB (N = 40), a smaller value of Rg appears (Rg = 1.29 nm), indicating that the polymer chain is in a larger shrinkage state. This is mainly because the steric hindrance effect of surfactants adsorbed onto polymer is less [42]. Surfactant and polymer mixtures tend to form a spherical aggregate (K^2^ = 0.026). K^2^ indicates the relative shape anisotropy. With the increase in surfactant concentration (N ≥ 50), the values of Rg become larger (1.42 nm ≤ Rg ≤ 1.65 nm), which means that the HPAM chain is more stretched. This is mainly attributed to the increase in steric hindrance. Surfactant and polymer mixtures tend to form rod-like aggregates (0.101 ≤ K^2^ ≤ 0.147). It can be seen that Rg in the equilibrium stage have a similar changing trend with K_2_ at different concentrations of DTAB.

These studies not only reveal the dynamic information of the polymer–surfactant self-assembly process at the molecular level but also provide an important theoretical basis for the application expansion of polymer/surfactant hybrid systems. An in-depth understanding of these self-assembly behaviours is of great practical significance for the development of novel materials and the optimization of industrial processes.

In summary, molecular modelling techniques play an important role in the study of polymer/surfactant self-assembly. Research in this field aims to reveal the mechanisms of polymer–surfactant interactions, as well as their structures and properties during the self-assembly process. This research also provides strong support for researchers to design and optimize self-assembly systems.

### 3.4. Molecular Simulation Applied to Acrylamide Cross-Linked Polymers

Cross-linked polymers are solution or gel systems with a three-dimensional network structure. In these systems, the crosslinking agent induces interactions between the polymer molecular chains through chemical bonding or physical entanglement, ultimately building a stable three-dimensional lattice system. Liu [44] used cross-linked polyacrylamide-filled osmotronic tensiometers, which are stable over long periods of time, in laboratory soil measurements. If further research is needed, molecular modelling is a better option. Measurement of soil suction becomes critical when exploring slope stability and soil-structure interaction issues associated with unsaturated soils beneath transportation infrastructure. Osmotic tensiometers (OTs) are capable of measuring soil suction in excess of 1000 kPa, but they face problems with pressure decay. By filling osmotic tensiometers with crosslinked polyacrylamide, stable pressure maintenance for longer periods of time can be achieved; however, research on this type of osmotic tensiometers is relatively scarce. Liu experimentally investigated the behaviour of synthetic nonionic crosslinked polyacrylamide-filled OTs and elaborated on them using the Flory–Huggins polymer theory. He also proposed a generalized calibration equation designed to reduce the effect of temperature changes on OT pressure readings. In the study of acrylamide crosslinked polymers, molecular simulation techniques are emerging as a new field full of potential and promise. The application of molecular simulation techniques provides a powerful tool for studying acrylamide crosslinked polymers, which can help researchers gain a deeper understanding of the microstructure and interaction mechanisms of crosslinked polymers at the atomic level. Through simulation, the cross-linking process can be accurately designed and optimized, and the macroscopic properties of cross-linked polymers can be predicted. This provides theoretical guidance and parameter optimization suggestions for experiments. In addition, molecular simulations can reveal the behaviour of crosslinked polymers under different environmental conditions, such as their performance in applications like oilfield water plugging, water treatment, and drug delivery. These studies not only help develop crosslinked polymer materials with better performance but also promote the innovation and improvement of these materials in industrial applications. With the improvement of computational power and simulation methods, the role of molecular simulation in the study of acrylamide crosslinked polymers will become more and more important, which is expected to promote the in-depth development of the research in this field and ultimately achieve technological breakthroughs and the industrial upgrading of crosslinked polymers in multiple applications.

#### 3.4.1. Microstructure and Reaction Mechanism of Organic Cross-Linked Acrylamide Polymers: Combined Application of Molecular Simulation and XPS Technology

Acrylamide crosslinked polymers are a class of polymeric materials synthesized by crosslinking reactions, which are capable of forming three-dimensional network structures that exhibit excellent strength and stability. The combination of molecular simulation with experimental techniques is becoming increasingly important in research in this field. Ni et al. [45] from Sichuan University investigated the para-condensation reactions between PAM and water-soluble phenolics using a combination of molecular simulation and X-ray photoelectron spectroscopy (XPS) techniques. In the study, they constructed a network model of crosslinked polyacrylamide (PAM) and quantified the number of active crosslinking sites and the density of crosslinked PAM by molecular simulation. The simulation results showed that the density of the product decreased after crosslinking, a phenomenon attributed to the formation of a supportive network between the PAM molecular chains by trimethylolphenol (THMP) during the crosslinking process, which led to a looser arrangement of the amorphous polymer molecular chains. In addition, it was found that the amide groups involved in the crosslinking reaction accounted for approximately 60% of the total amide groups in the PAM/THMP system, and the number of crosslinking points of PAM/THMP was higher than that of THMP/THMP. The results of structural characterization of the crosslinking products by the XPS technique are in agreement with the molecular simulation results, confirming that the condensation of amide groups is the main kinetic process in the reaction. In this process, the total number of reacted amide groups accounts for 61% of the total amide groups. However, the combination of molecular modelling and X-ray photoelectron spectroscopy (XPS) techniques has some obvious advantages and potential drawbacks when investigating the polycondensation reaction between polyacrylamide (PAM) and water-soluble phenolics: molecular modelling can provide dynamic and structural information at the molecular level, while XPS technology can provide information about the chemical state of a material’s surface. The combination of the two can provide a more comprehensive analysis. Molecular simulation can reveal the micro-mechanisms of the reaction, including reaction pathways, energy changes, intermolecular interactions, etc., which can help to gain a deeper understanding of the kinetics and thermodynamics of the polycondensation reaction. XPS technology can provide information on the elemental composition, chemical state, and valence band structure of the material surface, which is particularly useful for studying the interfacial reaction between PAM and water-soluble phenolics. The disadvantages are that molecular simulations require significant computational resources, especially for complex systems or large-scale molecular dynamics simulations, and that molecular simulations rely on theoretical models and assumptions, the accuracy of which directly affects the reliability of the simulation results.

This study not only reveals the microscopic mechanism of the cross-linking reactions of PAM gels but also has important guiding significance for the development of new PAM-based gel materials. An in-depth understanding of the kinetic process of these cross-linking reactions and the formation of the network structure has significant scientific value and practical application prospects for optimising the properties of the materials and expanding their applications.

#### 3.4.2. Molecular Simulations Reveal Interaction Mechanisms in Inorganic Cross-Linked Acrylamide Polymers

**Figure 6:**
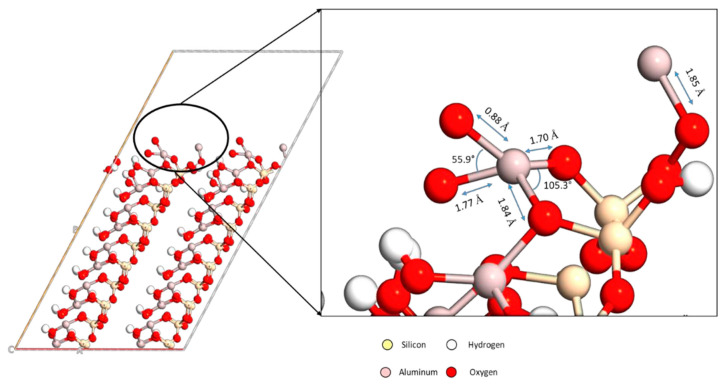
The optimized structure of two Al^3+^ ions adsorbed atop a clay surface. The image is repeated in the Y-direction for better clarity of the added aluminium ion with the aid of periodic boundary condition (PBC) [46].

In addition to its application in organic crosslinked polymer systems, molecular modelling techniques have been extended to the study of inorganic crosslinked polymer gel systems. Hamza et al. [46] from Qatar University crosslinked polyacrylamide (PAM) with aluminium acetate (AlAc) and introduced bentonite as an additive in their study, aiming to enhance the stability of the gel system. They investigated in detail the effects of bentonite addition and AlAc particle size on the PAM/AlAc gel system and deeply explored the interaction mechanism between the crosslinker and bentonite by molecular simulation. The experimental results revealed that when the content of bentonite is controlled at a low level of 0.5% to 1.0% (wt%), it can effectively retard the crosslinking reaction of AlAc with small particle size, and at the same time it has little effect on the strength of the gel. Through simulations, the scholars also evaluated the parameters related to the adsorption of Al^3+^ on the bentonite surface and found that the adsorption energy of a single Al^3+^ was 11.854 electron volts (eV). When a second Al^3+^ was introduced into the system, the adsorption energy decreased to 9.25 eV due to the mutual repulsion of the charges between the Al^3+^ ions. The adsorbed Al^3+^ on the bentonite surface hindered the interaction between AlAc and PAM, thus prolonging the gelation time. The reduction in question can be attributed to the electron repulsion that occurs among like-charged aluminium ions, as depicted in Figure 6. This phenomenon is a result of the Coulombic forces that act between the similarly charged particles, leading to the observed decrease.

Overall, the molecular simulation technique can simulate the polymerization process of acrylamide cross-linked polymers, covering the reaction kinetics and the evolution of the molecular structure. By simulating the rate and mechanism of the polymerization reaction, researchers can optimize the polymerization conditions and precisely control the structure and properties of the crosslinked polymer. In addition, molecular simulation provides a powerful tool for an in-depth understanding of the microstructure, properties, and behaviour of polymers. It also offers a solid theoretical basis and scientific guidance for the design, development, and application of novel materials [47].

## 4. Prospects for Acrylamide Polymer Oil Filtration

Acrylamide polymers have promising applications in oil repulsion, especially in enhanced oil recovery. By synthesizing acrylamide polymers with different functional groups, it is possible to improve their performance in oil reservoirs, such as reducing the viscosity of thick oil and increasing wave volume. Due to the high temperature of deep oil wells, traditional polyacrylamide-based oil repellents are easily hydrolysed and degraded at high temperatures. Therefore, improving the high temperature resistance of polymers is an important research direction. By sulfonation modification of polyacrylamide and the addition of heat stabilizers, the stability and oil repellent effect of polymers can be enhanced in high-temperature reservoirs. Meanwhile, for the non-homogeneity of the reservoir after polymer drive, studying the main controlling factors and designing individualized development parameters can significantly improve the recovery rate. Optimization of water injection, fluid production, and chemical concentration is the key to improving the performance of composite drive in non-homogeneous reservoirs. The salt tolerance of polymers is equally important in highly mineralized reservoirs. Research and development of salt-tolerant acrylamide polymers can maintain their oil repulsion performance in high-salt environments. Polymer oil drive technology can be used in combination with other enhanced recovery technologies (such as thermal drive, gas drive, microbial oil recovery, etc.) to achieve better oil drive results. At the same time, numerical simulation technology can be used to simulate and optimize the polymer oil driving process, which can reduce the number of field tests, lower the cost, and quickly find the optimal oil driving scheme.

In summary, the application prospect of acrylamide polymers in the field of oil drive is positive, but further research and development is still needed in the areas of high temperature resistance, salt resistance, environmental friendliness, and economy. Through continuous technological innovation and optimization, acrylamide polymers are expected to play a greater role in improving oil recovery.

## 5. Conclusions

High polymerisation acrylamide-based polymers are widely used in the oilfield due to their excellent properties. The application of molecular simulation allows us to probe deeply into the microstructure and size change mechanisms of these polymer systems, providing a powerful tool to understand the molecular structure, physical properties, and chemical behaviour of polymers. Through simulation, we can observe the structural features, dynamic behaviour, and intermolecular interactions of highly polymeric polymers at the nanoscale—information that is extremely valuable for polymer synthesis, design, and application development. Nevertheless, molecular simulation studies of high-polymerization acrylamide polymers still face a number of challenges. The size and structural complexity of polymers result in simulations that consume significant computational resources and time. In addition, for certain complex chemical reactions and kinetic processes, existing simulation methods may have limitations, and the accuracy of predictions could be improved. In the face of the continuous advances in oilfield development technology, the use of molecular simulation techniques to guide experimental studies will become an important direction for future research. This includes the rational design of polymer molecules, the prediction of their application effects, and the in-depth study of the microscopic mechanism of polymer action. Most molecular simulations rely on empirical or semi-empirical force fields to characterize interatomic interactions. These force fields are usually obtained based on experimental data or quantum chemical calculations, but they may not fully capture all types of chemical bonds and interactions, especially for complex or non-traditional chemical systems. Although molecular dynamics simulations can cover time scales from nanoseconds to microseconds, these time scales may still be insufficient for some very slow chemical reactions, such as catalytic or solid-state reactions. In order to achieve these goals, future molecular simulation studies should select appropriate tools and consider using a combination of simulation software to take advantage of their respective strengths. For example, software such as LAMMPS (lammps-16Mar18), GROMACS (Gromacs-5.1.2), and NAMD (NAMD 2.10) can be used for the simulation and visualization of molecular structure and kinetic behaviour; at the same time, software such as Gaussian (Gaussian 16), VASP (VASP6.3.2), and NWChem (NWChem 7.2.2) can be used to calculate the electronic structure, energy, and chemical reaction properties of molecules. Some software is listed and their advantages and disadvantages can be seen in Appendix A (Table A2). With the continuous progress and innovation of molecular simulation technology, it is expected that the systems that can be simulated in the future will be more complex and diverse, and the accuracy of the simulation results will be significantly improved. The application of molecular simulation in the oilfield field is promising and is expected to play a more critical role in the development of polymer oil drive technology.

## Data Availability

Not applicable.

## Appendix A

**Table A1:** Advantages and disadvantages of simulation methods.

Abbreviated	Interpretation	Advantages	Disadvantages
MD	Molecular Dynamics	MD simulation can be seamlessly integrated with a variety of molecular simulation methods, such as molecular docking and pharmacophore, to provide rich analytical tools.	Classical MD simulations must pre-construct the molecular force field of the simulated system; without the correct molecular force field, the simulation results will not correctly reflect the actual behaviour and properties of the simulated system.
MC	Monte Carlo	Through a large number of random sampling and simulation tests, very accurate results can be obtained, especially for complex high-dimensional problems and nonlinear problems, where the advantages are more obvious.	The speed of convergence of Monte Carlo simulations is usually related to the dimensionality and complexity of the problem.
CGMD	Coarse-grained molecular dynamics	Coarse-grained models can significantly improve the speed of simulation by reducing the number of particles in the system, increasing the computational efficiency by at least 3 to 4 orders of magnitude compared to all-atom simulations.	For some small molecules or specific types of chemical processes, coarse-grained models may not be accurate enough and all-atom models may be necessary.
BD	Brownian Dynamics	BD simulations can simplify the model by treating the solute as a rigid body moving in a continuous medium, ignoring the explicit representation of solvent molecules.	Solvent molecules are treated implicitly in BD simulations, which may ignore some important effects of solvent molecules, such as the dynamic structure of solvents and specific solute–solvent interactions.

**Table A2:** Advantages and disadvantages of simulation software.

Software	Advantages	Disadvantages	Whether or Not a Fee Is Paid
GROMACS (Gromacs-5.1.2)	Supports multiple levels of parallelism, including ensemble-level parallelism, MPI-level parallelism, and OpenMP multi-threading mechanism to fully utilize system parallelism.	While GROMACS is very powerful in biomolecular simulation, it may not be as feature-rich as other software such as LAMMPS in some specific areas, such as solid and materials science.	Free
LAMMPS (lammps-16Mar18)	LAMMPS supports the simulation of million-atom molecular systems in gaseous, liquid, or solid phases and provides good parallel scalability.	LAMMPS may not be as intuitive and convenient as some software with graphical user interfaces for complex modelling and key length control, etc.	Free
AMBER (AMBER 22)	AMBER offers a wide range of force fields to support different molecule types, including the universal force field GAFF for organic small molecules.	AMBER may be slightly less fast and flexible than some other molecular dynamics software.	Charging
CHARMM (CHARMM-Free Version 46b2)	Supports basic molecular dynamics algorithms, including multiple coefficients, multiple time steps, periodic boundary conditions, simple normal mode analysis, energy minimization, etc.	Due to the loose combination and the relatively independent development model, the software volume and complexity are increasing, which brings certain difficulties for maintenance and performance optimization.	Sectional fee
Materials Studio (Materials Studio2024)	Materials Studio has an intuitive graphical user interface that makes model building, parameterization, and visualization of results quick and easy.	During the calculation process, the results of Materials Studio may deviate from the experimental values to a certain extent, so it is necessary to objectively judge the availability of the data when using it.	Charging
